# Case report: Emergency management of unilateral herniation of bowel and a bilateral defect of the broad ligament in a resource limited setting

**DOI:** 10.1016/j.ijscr.2024.109269

**Published:** 2024-01-15

**Authors:** Anusha Ashkar, Fatima Zulfiqar Siddiqui, Arsalan Baig

**Affiliations:** Dow University of Health Sciences, Karachi, Pakistan

**Keywords:** Broad ligament herniation, Internal hernia

## Abstract

**Introduction:**

Broad ligament herniation is a rare type of internal hernia, presenting as a diagnostic challenge. An exploratory laparotomy can be performed to definitively diagnose and treat the defect.

**Case presentation:**

A 36-year-old female, with no known comorbids and no significant past medical and surgical history presented with acute abdominal pain, multiple episodes of vomiting, and absolute constipation. After clinical and radiologic investigations, the diagnosis of an internal herniation of small bowel to the broad ligament was suspected. An emergent exploratory laparotomy was then performed. Intra-operatively it was found that she had a bilateral defect to the broad ligament and a unilateral broad ligament hernia (BLH). Postoperatively she remained vitally stable and was discharged home.

**Clinical discussion:**

We report a rare case of a unilateral broad ligament hernia with a bilateral defect. Diagnosis of BLH is associated with diagnostic uncertainty mainly due to its rarity and nonspecific presentation.

**Conclusion:**

Although broad ligament herniation is rare, it is a significant cause of intestinal obstruction and may result in complications if not attended to timely. Early diagnosis and management is necessary to minimize associated morbidity and mortality that can occur as a consequence of bowel ischemia and necrosis.

## Highlights

• Broad ligament herniation is a rare type of internal hernia.
• It presents as a diagnostic challenge due to its vague presentation and rarity.
• Workup includes bloodwork (baselines) and imaging (X-rays and CT scans).
• Management is with emergent abdominal surgery, laparoscopic or open exploratory laparotomy.

## 1 Introduction

Internal hernia is defined as protrusion of abdominal viscera through peritoneal or mesenteric apertures into a compartment in the abdominal and pelvic cavities; internal hernias present as a cause in less than 10 % of the 350,000/annum cases of acute intestinal obstruction reported in the USA [1]. Broad ligament hernia (BLH) is a rare internal hernia, occurring in only 4–7 % of all reported cases of internal hernias [2]. The rarity of BLH presents as a diagnostic challenge. CT remains as the imaging tool of choice; however, diagnostic uncertainty remains until surgical intervention, as the only therapeutic modality of choice [2]. The present case shows that exploratory laparotomy can be safely performed to diagnose and repair unilateral BLH and a bilateral broad ligament defect. This case report conforms to the SCARE criteria [3].

## 2 Case presentation

A 36-year-old female with no known comorbidities, with no medical or surgical history presented to our teaching hospital with recurrent symptoms of intermittent abdominal pain and constipation which were managed conservatively over the past two decades. However, her current presentation was marked by acute symptoms, including:

• Abdominal pain for four days.
• Multiple episodes of vomiting for two days.
• Absolute constipation for two days.

The patient has had a history of three normal vaginal deliveries. Upon admission, her vital signs were recorded as follows: a pulse of 110/min, blood pressure of 120/90, afebrile temperature, respiratory rate of 25/min, and maintained normal oxygen saturation on room air. Physical examination revealed a distended abdomen, mild tenderness, and sluggish gut sounds. A digital rectal examination (DRE) revealed a collapsed rectum with no fecal staining. Laboratory data demonstrated normal full blood count, renal functions, and electrolytes.

**Fig. 1:**
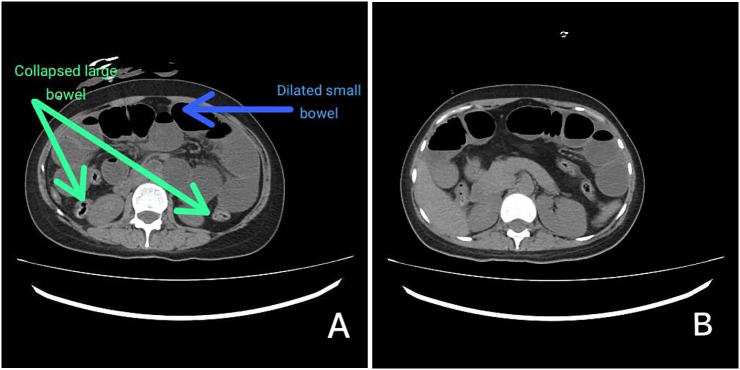
A and B. Non-contrast CT scans showing dilated bowel loops and collapsed large bowel.

Initial ultrasound imaging disclosed multiple fluid-filled bowel loops with sluggish peristalsis and a slender streak of inter-bowel fluid. Subsequently, a CT scan was performed, suggesting a dilated small bowel with a potential stricture or band in the terminal ileum due to inflammation (Fig. 1A and B).

**Fig. 2:**
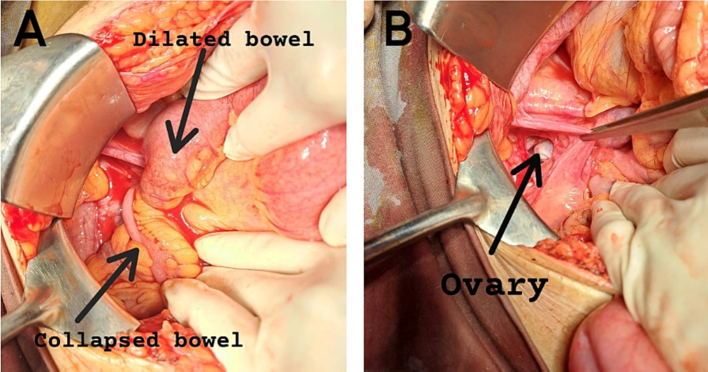
Intraoperative images showing dilated and collapsed bowel.

There was also mild free fluid in the abdominopelvic cavity, along with a collapsed large bowel (Fig. 2).

Diagnosis of acute intestinal obstruction was made and to address the patient's condition, a nasogastric tube (NGT) was inserted for decompression, and she received intravenous (IV) fluids as per the ‘drip and suck’ protocol.

After a thorough explanation of her diagnosis and the necessity for surgery, she provided written and informed consent.

An exploratory laparotomy was performed, during which 350 ml of ascitic fluid was drained. It was discovered that a 5 cm segment of the small bowel, located 1.5 ft proximal to the ileocecal junction (ICJ), had herniated internally through a defect in the (Cilley type-III defect) right-sided broad ligament. A similar defect (Cilley type-III) was found on the left-sided broad ligament however, no bowel herniation was found. Fortunately, the bowel was found to be healthy, the herniated bowel segment was carefully reduced, and the defects were repaired with Vicryl 2.0.

Following the successful surgical intervention, the patient remained vitally stable and was discharged home.

## 3 Discussion

Only 4 % of internal hernias are reported as broad ligament hernias [2,4]. The rarity of BLH occurrence itself presents a diagnostic challenge and leads to skepticism regarding a standardized treatment strategy.

Our case report is the first to report unilateral BLH and a bilateral broad ligament defect in Pakistan, a Southeast Asian country hence illustrates the diagnostic and treatment strategy of BLH in a resource-limited setting.

BLH is defined as an abnormal defect in the broad ligament leading to protrusion of mainly the small bowel; the causes of BLH can be categorized as either congenital or acquired which may include previous abdominal surgery, trauma, multiparity, and trauma during delivery, which may occur unilaterally or bilaterally, however, bilateral occurrence is rarely reported [4,5]. Our patient most probably presented with BLH secondary to unknown trauma in her sequential vaginal deliveries or as a congenital hernia.

Since the first reported case of BLH in 1861 by Quain, various classifications have been proposed [6]. The classification presented by Cilley et al. based on the anatomical defects are broadly classified into three subcategories: Type 1 defect caudal to the round ligament; Type 2 defect over and above the round ligament; Type 3 defect between the round ligament and the remaining broad ligament, through the mesoligametum teres [7]. Our patient is present with Type-III according to the Cilley classification [7].

The diagnostic uncertainty of BLH is reasoned due to the rarity of occurrence, non-specified symptoms, and laboratory tests. Since most of the cases present with either intestinal obstruction or bowel ischemia, the pre-operative definite diagnosis of BLH remains uncertainty.

Radiological imaging including ultrasound may reveal multiple fluid-filled bowel loops, however, does not lead to a definitive diagnosis of BLH.

CT scans, however, have emerged as the most favorable imaging modality for BLH [8]. CT findings include (i) dilated small-bowel loops with air-fluid levels in the Douglas pouch; (ii) distended loops pushing against the adjacent structures; and (iii) mesenteric vessels of herniated loops penetrating the broad ligament may be seen. In this case, all three characteristics were found.

Definitive treatment of BLH is surgical however, there are varied opinions regarding the surgical approach, laparoscopy, or explorative laparotomy.

Recent studies have suggested laparoscopic technique is associated with better post-operative outcomes and a shortened hospitalization period [9], however, in certain circumstances, laparotomy becomes the procedure of choice such as dilated bowel loops greater than 4 cm, inflammatory bowel disease, dense adhesions, and bowel ischemia [10].

In our case, we opted for exploratory laparotomy due to suspected bowel ischemia, diagnostic uncertainty, and limited workspace due to dilated bowel loops, to rule out the presence of other internal hernias, and to explore the contralateral broad ligament for any hernias and repair on the operative table.

It is pertinent to mention that laxity of ligament, specifically broad ligament in focus, can lead to untoward consequences such as prolapse and unusual hernias, due to this reason a laparoscopic approach is preferred to minimize said consequence [[11], [12], [13]].

## 4 Conclusion

BLH although rare, may lead to bowel ischemia and other associated complications if left unattended, hence surgery must not be delayed decreasing the chances of associated morbidity and mortality. Among the causes of bowel obstruction, BLH should always be ruled out, especially in patients with no surgical history or history of trauma.

## Ethical approval

This case report is observational in nature and is given exemption by the ethics committee of Dow University of Health Sciences.

## Funding

None.

## Guarantor

Dr Arsalan Baig and Dr Anusha Ashkar.

## Research registration number

Not the first case report to be reported about broad ligament herniation.

## CRediT authorship contribution statement

**Anusha Ashkar:** Conceptualization, Supervision, Validation, Writing – original draft, Writing – review & editing, Formal analysis, Investigation, Methodology, Project administration. **Fatima Zulfiqar Siddiqui:** Resources, Writing – original draft, Writing – review & editing, Formal analysis, Funding acquisition, Investigation, Methodology, Project administration. **Arsalan Baig:** Conceptualization, Resources, Validation, Writing – review & editing, Formal analysis, Investigation, Methodology, Project administration.

## Declaration of competing interest

None.
